# Computational Methods for Single-Cell Proteomics

**DOI:** 10.1146/annurev-biodatasci-020422-050255

**Published:** 2023-04-11

**Authors:** Sophia M. Guldberg, Trine Line Hauge Okholm, Elizabeth E. McCarthy, Matthew H. Spitzer

**Affiliations:** 1 Department of Otolaryngology–Head and Neck Surgery and Department of Microbiology and Immunology, University of California, San Francisco, California, USA;; 2 Biomedical Sciences Graduate Program, University of California, San Francisco, California, USA; 3 Gladstone-UCSF Institute for Genomic Immunology, San Francisco, California, USA; 4 Helen Diller Family Comprehensive Cancer Center, University of California, San Francisco, California, USA; 5 Institute for Human Genetics; Division of Rheumatology, Department of Medicine; Medical Scientist Training Program; and Biological and Medical Informatics Graduate Program, University of California, San Francisco, California, USA; 6 Parker Institute for Cancer Immunotherapy, San Francisco, California, USA; 7 Chan Zuckerberg Biohub, San Francisco, California, USA

**Keywords:** computational methods, mass cytometry, spatial proteomics, data analysis, clustering, trajectory inference

## Abstract

Advances in single-cell proteomics technologies have resulted in high-dimensional datasets comprising millions of cells that are capable of answering key questions about biology and disease. The advent of these technologies has prompted the development of computational tools to process and visualize the complex data. In this review, we outline the steps of single-cell and spatial proteomics analysis pipelines. In addition to describing available methods, we highlight benchmarking studies that have identified advantages and pitfalls of the currently available computational toolkits. As these technologies continue to advance, robust analysis tools should be developed in tandem to take full advantage of the potential biological insights provided by these data.

## INTRODUCTION

New technologies for high-dimensional protein quantification in single cells have spurred the development of analytical methods to maximize the insights that can be extracted from these datasets. Historically, flow cytometry has enabled proteins to be quantified in single cells using antibodies conjugated to fluorescent dyes as reporters. While the spectral overlap of these fluorophores limited the number of proteins that could be quantified simultaneously (generally 10–15 parameters), many foundational data analysis methods were nevertheless pioneered using these data (1). The development of mass cytometry by time-of-flight (CyTOF) increased the dimensionality of single-cell protein measurements to 40–50 parameters per experiment (2, 3), prompting the development of accompanying computational approaches for harnessing this increased information space. Recent advances in spectral flow cytometry and sequencing-based approaches leveraging antibodies tagged with oligonucleotides have utilized this analytical tool kit as well (4, 5).

While the aforementioned approaches measure protein expression in single cells in suspension, a recent technology boom has resulted in new methods for multiplexed spatial analyses of intact tissues as well. Multiplexed immunofluorescence approaches, mass-tagged antibody platforms, and oligonucleotide-based technologies are now broadly available (6–9). These techniques pose unique challenges and opportunities for the development of new computational biology approaches to maximize the potential of the spatial information encoded in the resulting data. Beyond multiplexed protein quantification in single cells, these techniques can also provide insights into the spatial arrangement of proteins and cells within tissues, requiring different classes of data analysis methods.

**Figure 1:**
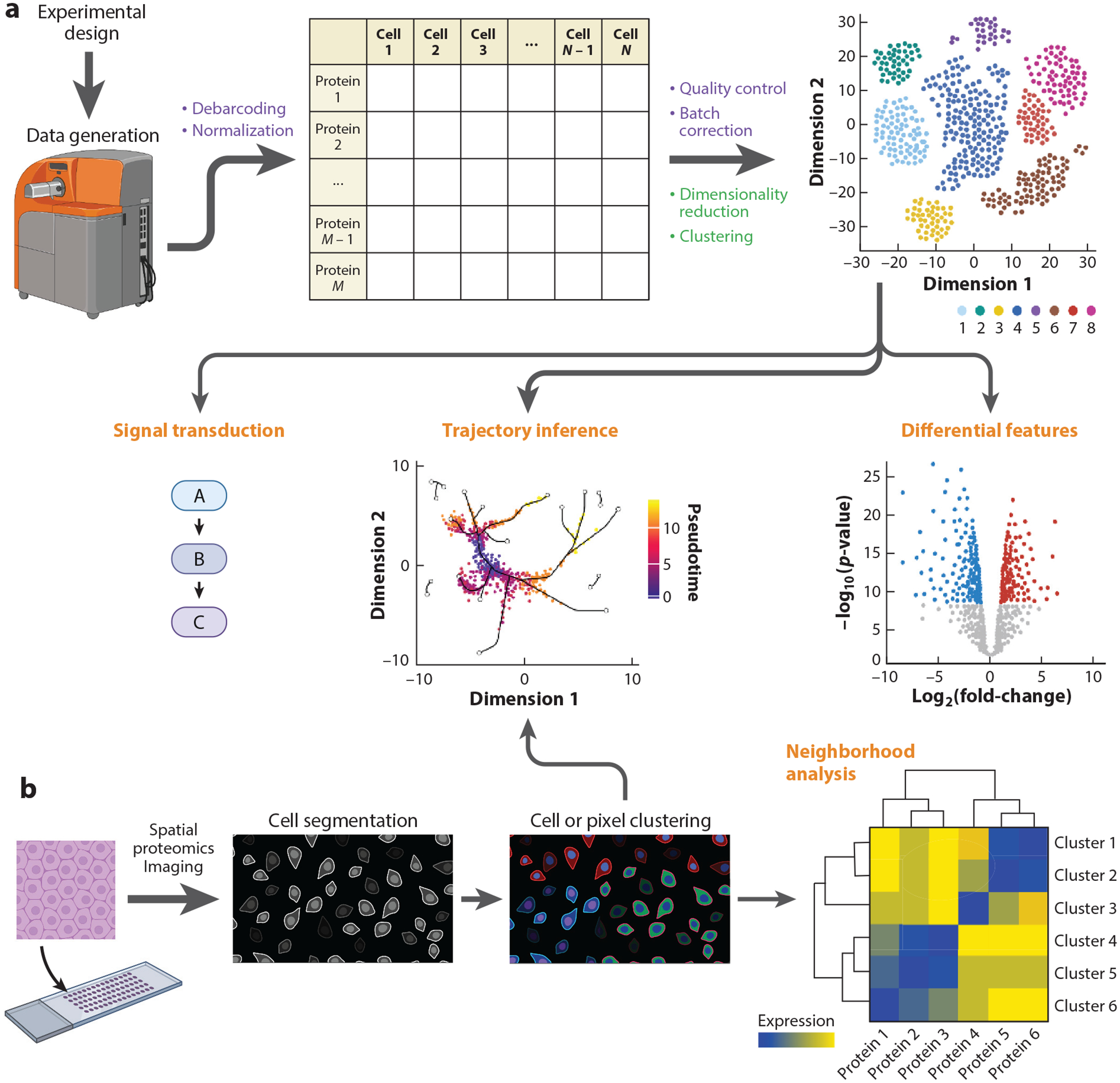
Overview of single-cell and spatial proteomics data generation and analysis. (*a*) Following proper experimental design, single-cell proteomics data are generated using a mass cytometer. Preprocessing steps (*purple*) include debarcoding and normalization to yield an *M* proteins × *N* cells expression matrix. After quality control and batch correction, the first steps of downstream analysis (*green*) are usually data visualization through dimensionality reduction and clustering. Later dataset-specific analysis steps (*orange*) might include differential feature analysis or trajectory inference. Single-cell proteomics datasets that include detection of posttranslational modifications often include signal transduction analysis. (*b*) Spatial proteomics uses a variety of techniques (our review focuses on multiplexed ion beam imaging by time-of-flight) to detect protein expression with spatial coordinates on arrayed tissue sections. Regardless of the data generation modality, cell segmentation is often the first analysis step after data preprocessing. Follow-up analysis often includes cell or pixel clustering and neighborhood analysis. Trajectory inference algorithms, either those that only use expression data or newer methods that also incorporate spatial information, can be used as well. Figure adapted from images created with BioRender.com.

In this review, we focus on the various classes of algorithms that have been developed for or applied to single-cell proteomics datasets across technology platforms (Figure 1). Each experimental technology requires data quality control, normalization, and other preprocessing steps. These single-cell data can be visualized with numerous dimensionality reduction algorithms, and clustering algorithms are commonly applied to partition cells that share similar multidimensional protein expression profiles. Statistical methods for differential cell abundances are commonly applied to identify biological differences across experimental conditions or groups of samples, as are methods to identify differential protein expression within cell populations of interest. Several trajectory inference algorithms can reconstruct differentiation or activation processes, leveraging the single-cell nature of these data. Spatial proteomics methods can generate single-cell protein imaging data within tissues to reveal cell–cell interactions and higher-order cellular neighborhoods. While these classes of methods have been applied across the single-cell proteomics technology landscape, here we particularly focus on mass cytometry and related imaging technologies, while the accompanying article in this volume by Fragiadakis and colleagues (10) addresses sequencing-based multiomics technologies, including protein quantification.

## DATA PREPROCESSING

The preprocessing steps of mass cytometry data are essential to ensure accurate results for downstream analyses and usually include parameter harmonization, bead-based normalization, debarcoding, pregating, and batch effect correction. Carefully planning an experiment can also improve the quality of the data for downstream computational analyses (see the sidebar titled Best Practices For Experimental Design).

### Parameter Harmonization

Since most analysis tools require identical panels across experiments, panel editing and renaming are necessary when combining data from separate experiments. Several methods, including cytofCore (https://github.com/nolanlab/cytofCore), cytutils (https://github.com/ismms-himc/cytutils), and Premessa (https://github.com/ParkerICI/premessa), enable antibody panel editing by removing unwanted channels, adding empty channels, or editing the isotope and antibody names. Additionally, Premessa can be used to concatenate multiple flow cytometry standard (FCS) files from a single CyTOF run.

### Bead-Based Normalization

Mass cytometry instrument performance varies over time due to decreasing detector sensitivity, built-up cellular debris, changes in plasma ionization efficiency, and manual interventions such as cleaning and calibration. Polystyrene beads are commonly used as internal standards for mass cytometry experiments (11) to limit the impact of technical variation. After adding beads containing different heavy metal isotopes to each biological sample, the median bead intensities can be calculated within a sliding window over time and across all samples in an experiment to normalize data for fluctuations in instrument sensitivity. Beads can be excluded from subsequent analysis steps based on a distance threshold from the centroid of the identified bead population. The bead standards normalization software was originally developed in MATLAB but has been reimplemented in R through Premessa.

### Debarcoding

Heavy metal barcoding is used to minimize technical variation by pooling samples together before antibody staining and data acquisition. The most common approach has been described by Zunder et al. (12). Briefly, cells from each individual sample are labeled with a unique combination of palladium isotopes before pooling, staining, and analyzing them using a mass cytometer. Running the barcoded samples together eliminates tube-to-tube variability in antibody staining and instrument performance. The barcoded samples are deconvoluted using single-cell debarcoding (SCD), in which barcode separation above a user-defined threshold is used to define positive and negative barcode channels for each individual cell. If the positive barcode channel combination corresponds to a sample defined in a barcode key, the cell is assigned to that sample. The SCD tool is available through Github (https://github.com/zunderlab/single-cell-debarcoder) (13) or Premessa.

### Data Transformation and Pregating

Mass cytometry data are usually arcsinh transformed for visualization and gating. This transformation compresses values in the upper end of the spectrum and enhances resolution in the lower end. The arcsinh transformation behaves similarly to a log transformation at high values but is approximately linear near zero and can accommodate zeros or small negative values, which can arise due to background subtraction and randomization of integer count values performed by default by the CyTOF software, as well as due to compensation for fluorescence cytometry data. For the transformation, a cofactor of 5 is usually used for mass cytometry data, while a cofactor of 150 is commonly used for fluorescence cytometry data to control the width of the linear region.

Pregating is performed to identify live single-cell events (singlets) in the data. FCS files can be uploaded to CellEngine (https://cellengine.com), Flowjo (https://www.flowjo.com), Cytobank (14, 15), or flowCore (16) to manually gate out debris, doublets, and dead cells. After bead-based normalization, any residual beads can be removed by gating events based on the intensity of a DNA intercalator reagent channel and an isotype channel that is contained within the beads. Beads will be DNA negative and bead isotope positive. Next, singlets are obtained by plotting a DNA channel against event length, which measures the number of mass scans that were integrated to identify each event in the data. Events that have a length within the range of the majority of events are enriched for singlets. Finally, dead cells are gated out based on the intensity of a viability stain (e.g., cisplatin). Since dead cells have disrupted membranes, cisplatin can enter these cells quickly and form bonds with intracellular molecules. Therefore, live cells are obtained by excluding cells with high amounts of viability stain. When preparing for downstream analyses, it is helpful to also isolate any specific cell populations of interest if relevant (e.g., immune cells).

Manual inspection of gates is always recommended, although this strategy can be time consuming and laborious for large experiments. Gates drawn on a few samples can be reproduced across entire datasets in common flow cytometry analysis software tools and manually tailored as necessary, or more automated approaches, such as openCyto (17), can also be used (18, 19).

### Quality Assessment

Samples should be inspected to evaluate overall quality. Manual gating can be used to evaluate marker expression across all samples and to identify specific markers with staining irregularities or abnormal signal changes that should be removed. Additionally, assessing the number of cells per sample and overall sample composition across cell types by multidimensional scaling (MDS) (20) or principal component analysis (PCA) (21) plots can also identify problematic samples that should be excluded from further analysis.

### Batch Effect Correction

**Figure 2:**
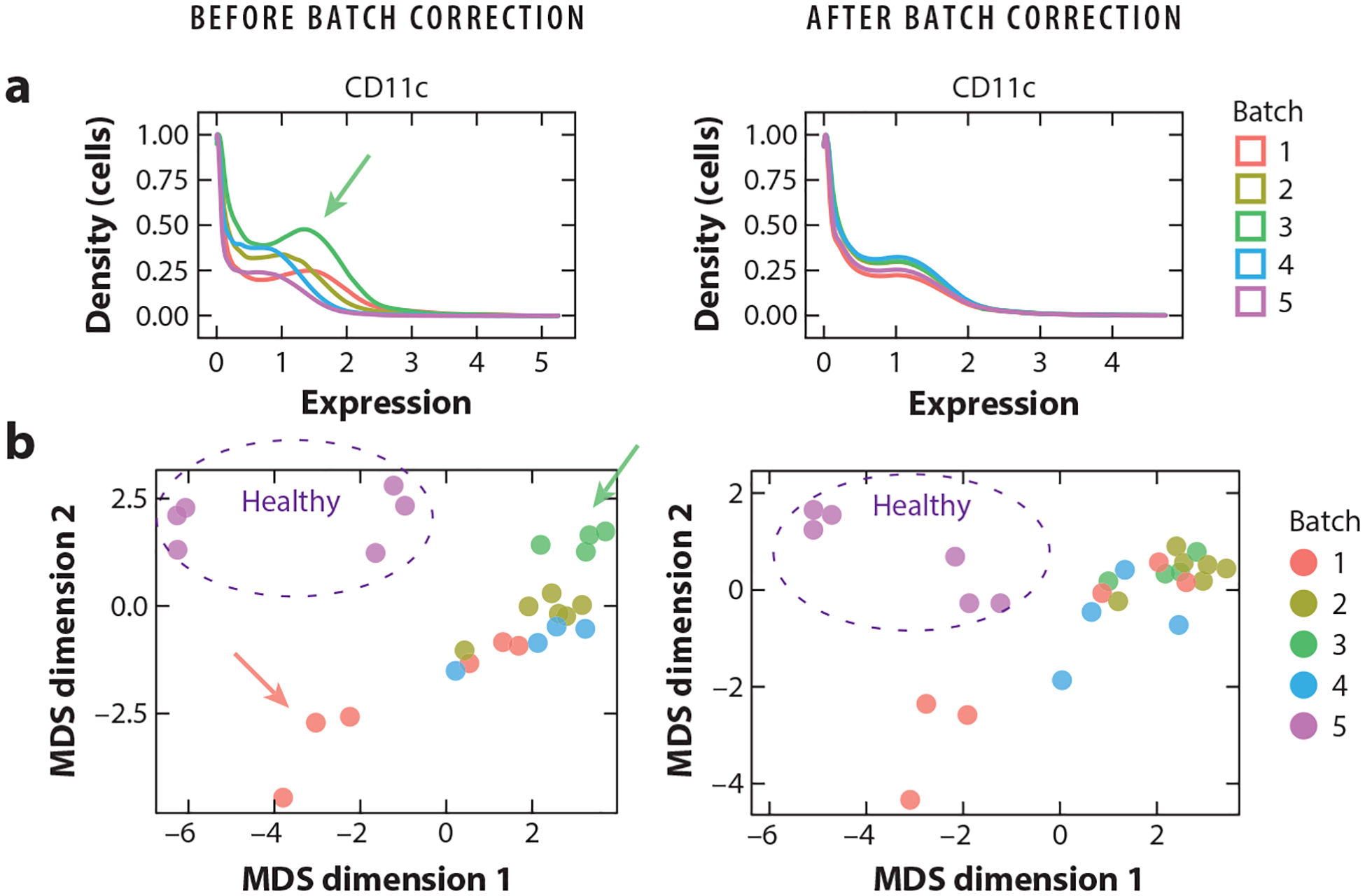
Correcting for batch effects in mass cytometry data. Here, samples have been obtained from COVID-19 patients (batches 1–4) and healthy individuals (batch 5) (102). All batches were run with a reference sample. Plots are generated before (*left*) and after (*right*) batch correction using CytoNorm. (*a*) Density distribution of CD11c expression in the reference sample replicated across the five batches. Before batch correction, the density distribution varies between batches (e.g., see *green arrow* pointing to batch 3). CytoNorm removes batch effects. (*b*) Multidimensional scaling (MDS) plots of human samples in batches 1–5. Before batch correction, samples are generally grouping according to batch (e.g., see *green arrow* pointing to samples from batch 3) or individuals (see *red arrow* pointing to three samples from the same individual on different days from batch 1). CytoNorm removes batch effects (*green dots* dispersed), while preserving biological differences (i.e., samples from healthy individuals in batch 5 are still grouping together, as are samples from the same individual from batch 1).

The last part of preprocessing is correcting for batch effects (Figure 2). While bead-based normalization addresses the technical variability between samples from the same experiment, batch effect correction is critical to remove variation between multiple runs and differences that are not captured by bead signals, such as differences in sample collection time, processing, or staining.

The biggest challenge for batch correction is to remove unwanted technical artifacts while preserving true biological signals. One solution is to include a shared reference sample across all batches in an experiment. Batch effects are captured as differences between the reference samples, and all samples within a batch are adjusted according to their reference sample. Several methods are built upon this approach, including CytofBatchAdjust (22), CytofRUV (23), and CytoNorm (24). While CytofBatchAdjust calculates a scaling factor for each marker per batch, CytoNorm identifies cluster-specific goal distributions per marker per batch, since technical variation can impact cell types differently. However, including a reference sample might not always be feasible, as in, for example, prospectively expanding clinical cohorts that indefinitely enroll patients. A single reference sample may not capture all potential sources of variability, such as when samples are collected at multiple sites. Additionally, if the samples of interest exhibit markedly altered immunophenotypes, such as samples obtained from patients with rare immunological disorders, all cell types might not be present in the reference sample. Therefore, other methods have been developed that perform batch correction independent of technical replicates.

iMUBAC (integration of multibatch cytometry datasets; 25) uses user-defined control samples in each batch to capture the average immunophenotype across controls. Cells from control samples across batches are then aligned using Harmony (26) before clustering. However, due to the intrinsic heterogeneity of human samples, single control samples across batches may not be biologically similar, which could complicate batch correction. The method cyCombine (27) enables the combined normalization of proteomics data from different batches, experiments, and technologies [e.g., CITE-seq (cellular indexing of transcriptomes and epitopes by sequencing), flow cytometry, and mass cytometry] by utilizing the empirical Bayes method. Batch correction is performed by standardizing expression values within each batch to enable clustering of all samples using a self-organizing map (SOM), followed by a per cluster normalization using ComBat (28). To enable multi-dataset analyses of experiments with different antibody panels, cyCombine includes a module for panel integration. Here, SOM clustering is performed on overlapping markers, and missing values are imputed from coclustered cells of the other panel. However, batch correction is only possible for markers present in all batches.

Choosing the right batch correction method depends on the experimental setup, the number of samples, and the inherent nature of the data. Several methods depend on clustering prior to normalization, including CytoNorm and CytofRUV, which assumes that batch effects do not heavily influence this initial clustering step. To circumvent this issue, cyCombine transforms markers within batches prior to clustering, and iMUBAC utilizes Harmony before clustering. Additionally, most methods assume that all cell subsets are found in all samples and that the reference/control samples resemble the other samples. If multiple tissue types are compared (e.g., blood and tumor), several reference samples could be incorporated, or investigators can spike in specific cell subsets that are lacking from the reference sample to capture the full diversity of expected cell types. Usability and run-time are also important to consider, especially for large studies. The methods described here are summarized in Table 1. They are all available as R packages, and a comparison of the tools is available in the paper reporting the cyCombine method (27).

Finally, with the rapid growth of mass cytometry data, integrating and analyzing public datasets become desirable. CytofIn (29) is a computational pipeline that enables integrated analyses of publicly available mass cytometry datasets. CytofIn uses regular expression matching to homogenize mass cytometry data files and generalized anchors, which are nonidentical references that exhibit low signal variability across datasets, for normalization.

## DIMENSIONALITY REDUCTION

Due to the large number of features profiled, data visualization and downstream analysis are dependent on transforming high-dimensional single-cell proteomics data to a low-dimensional space. Dimensionality reduction techniques can be subdivided into linear and nonlinear approaches.

The primary linear dimensionality reduction methods used are PCA, classical MDS, and independent component analysis. Linear methods such as PCA were commonly used with mass cytometry datasets when the technology was initially introduced (30), but currently, the majority of applications use nonlinear dimensionality reduction techniques in order to capture higher-order relationships among the input features.

**Figure 3:**
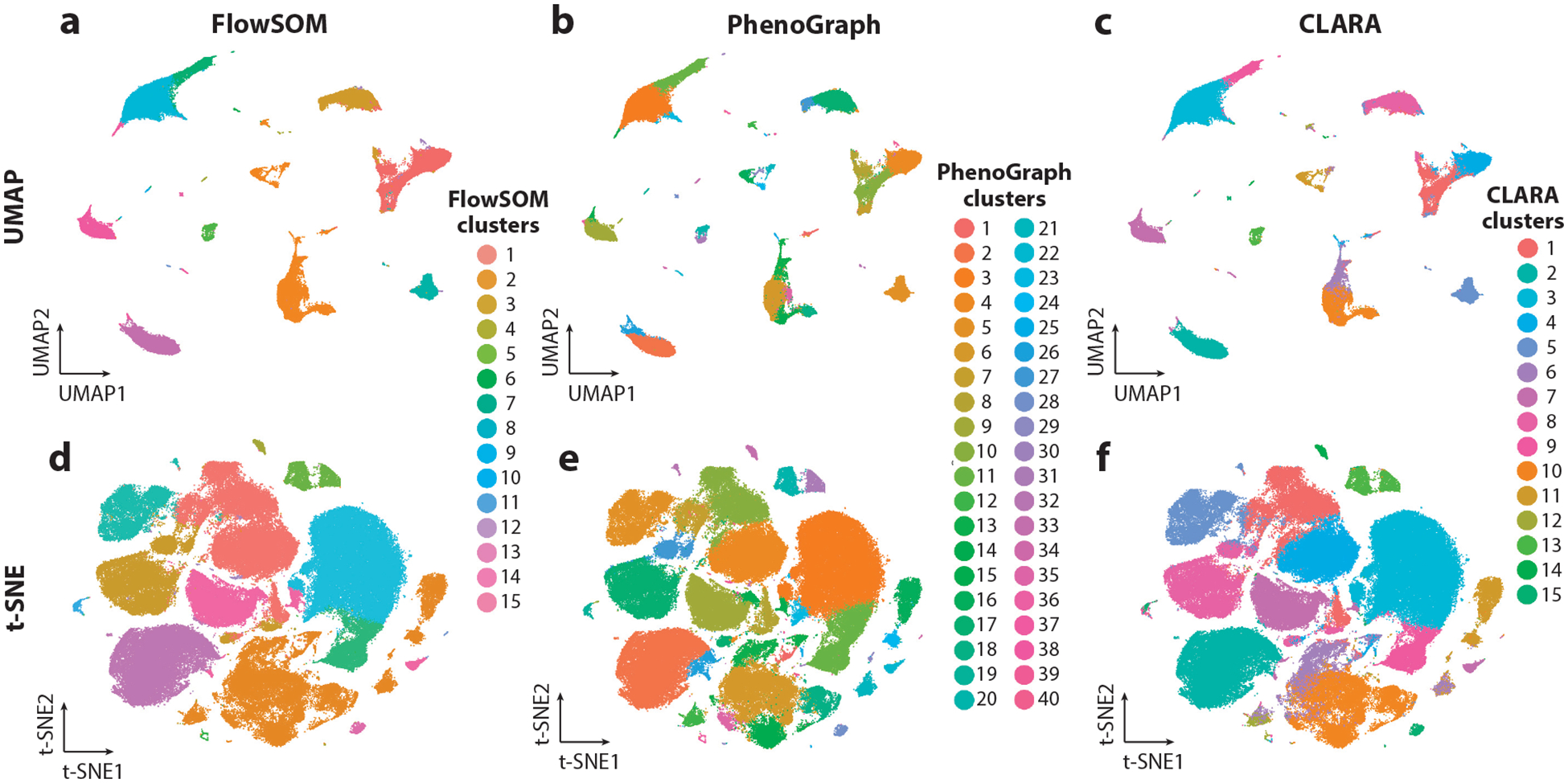
Common clustering algorithms and dimensionality reduction techniques for single-cell proteomics data. The sample shown represents 172,948 peripheral blood immune cells from a COVID-19 patient at a single time point (102). (*a*–*c*) UMAP (uniform manifold approximation and projection) dimensionality reduction colored using three clustering techniques: FlowSOM (self-organizing map) (*a*), PhenoGraph (*b*), and CLARA (Clustering Large Applications) (*c*). FlowSOM and CLARA require the number of clusters (*k*) to be specified, which was chosen here based on the expected number of immune populations. PhenoGraph requires the number of neighbors (here, the default value, *k* = 30, was used) to be specified rather than the number of clusters. (*d*–*f*) t-SNE (*t*-distributed stochastic neighbor embedding) dimensionality reduction colored using the same three clustering techniques as in panels *a*–*c*.

Nonlinear dimensionality reduction techniques vary in their ability to preserve global and local relationships in low-dimensional space. The package viSNE (31) implements a version of the nonlinear dimensionality reduction method *t*-distributed stochastic neighbor embedding (t-SNE) (32) tailored for mass cytometry data. However, while t-SNE performs well at preserving local structure, its poor performance at preserving global structure led to a shift in the field toward uniform manifold approximation and projection (UMAP) (33, 34), which better preserves both local and global structure (Figure 3). Other methods utilizing unsupervised machine learning such as SAUCIE (35), a neural network approach, and scvis (36), a generative model approach, have also been introduced. Recently, a benchmarking analysis of 20 dimensionality reduction techniques focused specifically on mass cytometry datasets (37) identified SAUCIE as the best overall performer along with a group of other top performers including nonclassical MDS, UMAP, scvis, PHATE (potential of heat diffusion for affinity-based transition embedding) (38), and t-SNE. Thus, broader adoption of dimensionality reduction techniques based on machine learning, such as SAUCIE, may lead to improved results. Wang et al. (37) also developed a package CytofDR (https://cytofdr.readthedocs.io/) that combines a variety of dimensionality reduction methods to enable easy application of multiple methods to a dataset.

## CLUSTERING

### Manual Gating Versus Unsupervised Clustering

As the dimensionality of single-cell proteomics datasets has increased, manual gating has remained a useful first step to enable high-level cell population separation using known markers. However, manual gating becomes increasingly laborious when dimensionality increases. Several semiautomated clustering methods have been introduced to address the increasing data complexity (Figure 3) (39). The introduction of unsupervised clustering methods has not only increased speed of analysis but also helped to reveal novel cell populations.

### Hierarchical Clustering

One of the most commonly used types of unsupervised clustering is agglomerative hierarchical clustering. This technique builds a dendrogram that initializes with a separate cluster for each cell and gradually merges cells into different clusters based on a distance metric to eventually encompass the entire dataset. A user-defined cutoff is used to determine the dendrogram level for creating cluster labels. Thus, the user must be able to approximate the number of desired cell populations to prevent over- or under-clustering the data. While this approach can be very useful for broad immune cell populations, it can risk missing rare cell types. Some commonly used hierarchical clustering methods include FlowSOM (40, 41), SPADE (spanning tree progression analysis of density normalized events) (42), and Rclusterpp (https://github.com/nolanlab/Rclusterpp).

FlowSOM is one of the most commonly used hierarchical clustering methods. The FlowSOM workflow relies on a SOM that is trained and visualized by a minimum spanning tree (MST) (40, 41). The lower-level SOM clusters are then subjected to a second, higher-level clustering to generate meta-clusters via consensus hierarchical clustering. FlowSOM has been evaluated against other nonhierarchical clustering methods such as ACCENSE (t-SNE-based clustering) (43), flowMeans (*k*-means clustering) (44), and flowClust (model-based clustering) (45) and has been found to perform with higher precision and faster runtime (46).

### Partitioning Clustering

Partitioning clustering subdivides the dataset into *k* groups according to each group’s center point. There are two common ways to define the center point: *k*-means clustering and *k*-medoids clustering (47). The former, *k*-means clustering, defines each cluster center as the mean and is more susceptible to outliers. The latter, *k*-medoids or partitioning around medoids (PAM), defines each cluster center as the medoid and uses a Manhattan distance metric rather than Euclidean distance, making it more robust to outliers but also more computationally intensive (48, chapter 2). Since non-Euclidian data metrics are much more computationally expensive, CLARA (Clustering Large Applications) (48, chapter 3) was introduced as an extension of the *k*-medoids approach and uses the sampling approach to handle large datasets (47, 49). SCAFFoLD (50) is one of the currently existing methods that utilizes CLARA clustering and landmark nodes (manually gated cell populations) to create a force directed connected graph visualizing cluster and landmark node relationships. Both *k*-means and medoid-based clustering approaches require the user to specify the number of desired clusters. Frequently, the elbow method is used to determine the optimal number of clusters, which is based on minimizing sum of squared distances between data points and their cluster centers with the fewest number of clusters (51).

### Community Network Detection

In community network detection, there are currently three popular algorithms used to analyze high-dimensional cytometry data: Louvain, Leiden, and PhenoGraph. The Louvain (52) algorithm initializes each cell as a separate node. Nodes are moved locally, then iteratively aggregated into larger communities based on the partition obtained in the local moving phase until maximum modularity is achieved. This results in a hierarchical structure without requiring the input of community size or number and enables analysis of large datasets due to its fast runtime (52). The Louvain algorithm’s reliance on the resolution limit of modularity and its iterative manner can sometimes result in poorly connected or disconnected communities, which led to the introduction of the Leiden (53) algorithm.

The Leiden algorithm is faster and more complex than Louvain and involves local moving of nodes, refinement of the partition, and aggregation of the network based on the refined partition (53). The Leiden algorithm does not require that the user specify any parameters, but the user can specify arguments for partition type and resolution to further tune the partitions and number of clusters.

PhenoGraph (54) incorporates the Louvain method and was designed for high-dimensional single-cell datasets. Distinct from the Louvain algorithm, PhenoGraph first uses Euclidean distance to find the *k*-nearest neighbors (KNN) for each cell and then builds a weighted graph (54). The Louvain algorithm is then used to maximize modularity. In PhenoGraph, the user must specify the number of nearest neighbors to be used for the KNN graph, which affects the number of clusters.

### Choosing the Best Clustering Algorithm

No clustering algorithm is perfect, and the best choice is often dependent on the dataset of interest. Comparisons of clustering methods on existing publicly available datasets have concluded that the correct clustering algorithm is dataset dependent, although some algorithms such as PhenoGraph and FlowSOM tend to perform better overall (46, 47, 55, 56). It is important to evaluate data for factors such as size, presence of outliers, dimensionality, and expected cell populations prior to choosing a clustering method. While most datasets do not have a ground truth, it is important to evaluate the performance of the chosen clustering algorithm compared to manual gating and other clustering algorithms and to assess the variation in output based on the choice of user-defined input values.

### DIFFERENTIAL FEATURES ANALYSES

After cell populations are obtained, by either manual gating or clustering, differential abundance and expression analyses are often used to identify differences between experimental groups. For single-cell proteomics data, differential abundance (DA) analysis can identify cell populations with changes in frequency between conditions. Differential expression (DE) analysis can identify differences in protein expression within cell populations. Various statistical tests (e.g., the nonparametric Wilcoxon Rank Sum Test) followed by an appropriate multiple testing correction can be used for comparing groups. In addition, some bioinformatics tools have been developed specifically for DA and DE analysis of single-cell proteomics data (see the sidebar titled Automating the Preprocessing Steps and Analysis Workflows).

### Differential Abundance Analysis

Some of the methods developed for DA analysis include Citrus (57), Statistical Scaffold (58), and CellCnn (convolutional neural network) (59). Citrus uses hierarchical clustering and regularized supervised learning algorithms to identify clusters and markers that are the best predictors of an outcome variable. Statistical Scaffold builds upon SCAFFoLD maps (50) and utilizes the Significance Analysis of Microarrays framework to identify features that are different between groups. CellCnn is optimized for analyzing rare cell populations by using convolutional neural networks to identify clusters that are associated with a specific phenotype.

However, these methods are unable to accommodate more complex experimental designs, such as longitudinal studies, paired data, or experiments with multiple factors and covariates (e.g., batch effects) without modification. Since cell counts roughly follow a negative binomial distribution, generalized linear models have been adapted for mass cytometry data with more complex experimental designs. Cydar (60) does not rely on an initial clustering step, but instead allocates cells into hyperspheres in a multidimensional marker space, and utilizes the negative binomial generalized linear model implemented in edgeR (61, 62) for DA analysis. Diffcyt (63) uses the FlowSOM clustering algorithm to identify cell clusters and includes implementations of various methods for differential testing, including linear mixed models, edgeR, limma (64), and voom (65). Additionally, cytoGLMM (generalized linear mixed model) (66) implements multiple regression that accounts for marker correlations on gated cell types. The performances of Citrus, CellCnn, cydar, and diffcyt are compared in the paper reporting the diffcyt method (63).

### Differential Expression Analysis

Analysis of marker expression is used to identify proteins that are differentially expressed between two groups within the same cell population. For example, diffcyt utilizes linear mixed models on median marker expressions within clusters. While summarizing marker expression across cells to a single value is the most common approach, this procedure assumes homoscedasticity and ignores other characteristics of the distribution, such as variance, bimodality, and skewness, as well as the number of cells within each cluster. Although median marker expression is generally informative to identify interesting changes, a model that takes the entire marker distribution into account, such as the Earth mover’s distance (67), would improve the ability to identify different cell states between groups.

### TRAJECTORY INFERENCE

Trajectory inference predicts cell alignment along a biological process such as differentiation. This inferred trajectory, or pseudotime, can be used to compare the distribution of samples and to interrogate which features change along the inferred trajectory. The majority of trajectory inference tools focus on single-cell RNA sequencing (scRNA-seq) datasets, but a subset of them have been specifically developed for mass cytometry datasets.

Trajectory inference algorithms can provide either a qualitative trajectory, which orders single cells or cell clusters in a graph-based format, or a quantitative trajectory with a pseudotime assignment for each cell. Here, we highlight nine algorithms that have been used with or were developed specifically for mass cytometry datasets. In Table 2, we also summarize metrics that can guide algorithm choice and highlight applications of each method.

### Graph-Based Algorithms

Diffusion map (68, 69) is a nonlinear dimensionality reduction method based on the random movement of cells. First, the random movement, or diffusion, in the high-dimensional protein expression space of each cell is modeled from its current protein expression. Then, the kernels, or covariance, of the models for each pair of cells are used to calculate a transition matrix. The eigenvectors of this transition matrix, called diffusion components, are used to visualize the cells.

PAGA (Partition-based Graph Abstraction) (56, 70) creates a connectivity graph of user-defined partitions, or clusters. The connectivity measure is based on the number of edges between cells in each cluster in a KNN-like graph compared to the expected number of edges with random edge assignment.

SPADE (3, 42, 71) outputs a connected tree of clusters of cells. First, the cells are downsampled to equalize density across rare and abundant cell subsets. The downsampled cells are clustered with an agglomerative hierarchical clustering algorithm into a user-defined number of clusters, and the resulting clusters are connected with an MST. Finally, each cell from the full dataset is mapped to the tree based on its nearest neighbor in the downsampled set.

### Pseudotime Algorithms

Among the algorithms that provide a cell pseudotime assignment, both Wanderlust (72) and SCORPIUS (73) can only detect linear trajectories. Wanderlust begins by creating an ensemble of KNN graphs and calculates pseudotime for each cell based on the shortest path to randomly chosen waypoint cells. The pseudotime for each cell is assigned as the average value over all the graphs. SCORPIUS maps cells using MDS based on the correlation matrix from the single-cell expression vectors. Next, pseudotime is calculated based on the shortest path (or principal curve) that connects cells within MDS space. Finally, a random forest model is used to interpret the proteins that drive the pseudotime ordering.

Other algorithms can detect more complex branched trajectories. CytoTree (74) uses a KNN graph to calculate pseudotime for each cell based on the distance from the cell to user-defined root cells. CytoTree can also be used to estimate intermediate state cells in branches using user-defined leaf, or terminally differentiated, cells. Monocle2 (75) uses an iterative method called reversed graph embedding to assign pseudotime. Each iteration includes four steps: initializing a dimensionality reduction, creating a spanning tree of centroids chosen by *k*-means clustering, shifting cells toward the nearest vertex, and mapping the tree (trajectory) back to the original high-dimensional space. Once the tree geometry and cell positions have converged, the pseudotime for each cell is assigned based on distance along the tree from a user-defined root cell. Slingshot (76) uses MST on user-defined clusters within the provided dimensionality reduction coordinates to define the trajectory path and branches. Simultaneously, the principal curves are optimized for each lineage. The pseudotime for each cell is given by the ordering of its orthogonal projection onto the principal curves.

Diffusion pseudotime (DPT) (56, 77) and Wishbone (78) are trajectory inference algorithms that detect complex trajectories based on diffusion maps. DPT uses a distance metric for each cell to a user-defined root cell based on the transition matrix, which was used to create a diffusion map, to calculate pseudotime. Wishbone first constructs a KNN graph based on a diffusion map. Similar to Wanderlust, iteratively random waypoint cells are chosen, with the addition of a refinement step to exclude outlier cells, and the trajectory position (or pseudotime assignment) for each cell is calculated based on the shortest paths to the user-defined root cell and the waypoint cells. Waypoint-dependent disagreements about the length of paths are used to identify branches of the trajectory. Iteratively, the branch identification and pseudotime assignments are updated until convergence.

### Algorithm Choice

Choosing the appropriate algorithm for mass cytometry trajectory inference involves many of the same considerations as scRNA-seq (79): (*a*) What is the expected type of trajectory in the dataset (e.g., linear, bifurcating, cyclic)? (*b*) Are the outputs of the algorithm qualitative (only graph based) or quantitative (pseudotime cell assignment)? (*c*) What are the required user-defined inputs, e.g., starting (i.e., root) cells?

In a recent benchmarking analysis (79) for trajectory inference methods, PAGA, SCORPIUS, and Slingshot were some of the best-performing methods across datasets with diverse types of expected trajectories, although only scRNA-seq datasets were analyzed. As recommended for scRNA-seq datasets (79), we suggest running two trajectory inference algorithms that can be easily implemented with dynverse (https://dynverse.org), a package that provides wrappers for most available trajectory inference algorithms for ease of use and comparison.

## SIGNAL TRANSDUCTION ANALYSIS

Another class of algorithms developed for single-cell proteomics data has focused on datasets in which activated cell signaling proteins are quantified within individual cells. By leveraging antibodies that specifically recognize posttranslational modifications on signaling proteins (often the phosphorylated forms), it is possible to infer how these pathways are activated in cells, either endogenously in vivo or upon stimulation with defined receptor ligands in vitro. Pioneering work from Sachs et al. (80) adapted Bayesian network approaches to reconstruct known T cell receptor signaling cascades from phospho-specific flow cytometry data in the context of various signaling inhibitors.

Krishnaswamy et al. (81) developed DREMI (Density Resampled Estimate of Mutual Information) and an accompanying data visualization technique (DREVI) to measure the relationships among signaling proteins in the same pathway. The approach quantifies the expression of a downstream signaling protein as a function of the expression level of an upstream regulator across single cells. When applied to T cell receptor signaling, the authors discovered that memory T cells require less input from upstream signaling proteins to achieve equivalent levels of phosphorylated downstream signaling proteins as compared to naïve T cells, consistent with the well-established reactivation potential of memory cells. Building upon this concept, Mukherjee et al. (82) developed flux-based modeling approaches to quantify signaling synergy between pathways, with applications to cytokine and NKG2D signaling in natural killer cells.

## SPATIAL PROTEOMICS ANALYSIS

Spatial proteomics technologies integrate spatial architecture with cell phenotype data, which has the potential to enhance understanding of disease progression and the development of therapeutics (83–87). Among the most commonly used spatial proteomics technologies are multiplexed ion beam imaging by time-of-flight (MIBI-TOF) (83), imaging mass cytometry (IMC) (88), and codetection by indexing (CODEX) (8).

**Figure 4:**
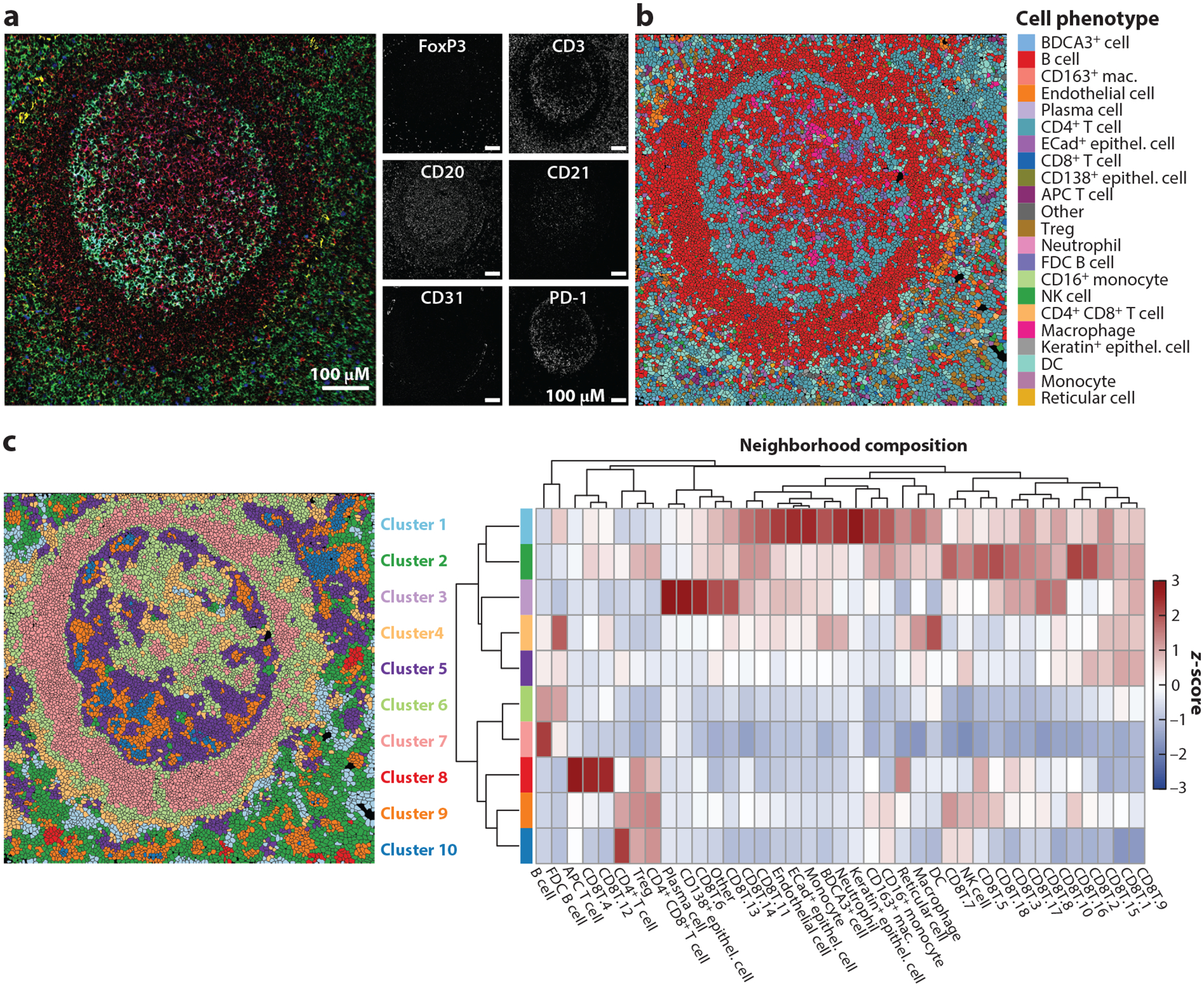
MIBI-TOF data analysis and visualization methods. The sample shown represents a lymph node from a human patient. (*a*) MIBI-TOF image with multichannel overlay (*left*) and single-channel images (*right*). (*b*) MIBI-TOF image overlaid with cell phenotype assignment from clustering. (*c*) MIBI-TOF image overlaid with *k*-nearest-neighbor analysis (*left*) and heatmap of neighborhood composition for each cluster/neighborhood (*right*). CD8^+^ T cells were subclustered, and decimals indicate different CD8^+^ T cell cluster numbers. Abbreviations: APC, antigen-presenting cell; DC, dendritic cell; ECad, E-cadherin; FDC, follicular dendritic cell; mac., macrophage; MIBI-TOF, multiplexed ion beam imaging by time-of-flight; NK, natural killer; Treg, regulatory T cell. MIBI-TOF images provided by Maha Rahim and Candace Liu.

MIBI-TOF and IMC both use heavy metal tagged antibodies to achieve highly multiplexed imaging but differ by their respective uses of an ion beam versus a laser to release ions from the tissue for quantification by time-of-flight mass spectrometry. Much like the advantages of mass cytometry over flow cytometry, these methods allow for interrogation of over 40 unique proteins (83). As newer technologies, analysis methods are still in active development, and important considerations for setting up data acquisition have been reviewed elsewhere (83, 89). The Angelo lab at Stanford has developed the toffy and ark-analysis pipelines for processing and biological interpretation of MIBI-TOF data (https://github.com/angelolab/toffy; https://github.com/angelolab/ark-analysis; Figure 4).

**Figure 5:**
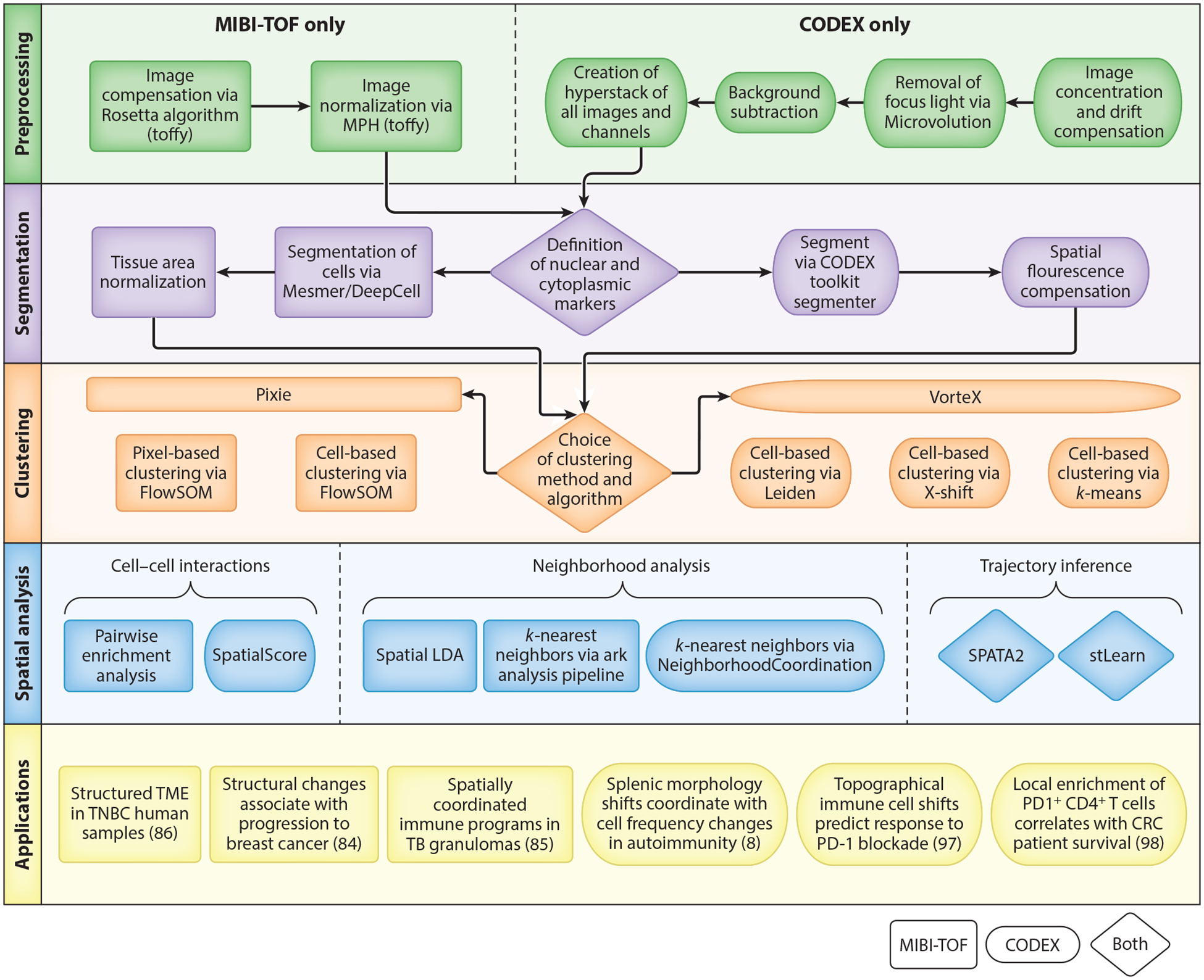
Flow chart depicting steps of MIBI-TOF and CODEX processing. Object shapes indicate the technology for which the method was developed. However, tools can be used across technology platforms, with the exception of those used for the preprocessing steps. Abbreviations: CODEX, codetection by indexing; CRC, colorectal cancer; LDA, latent Dirichlet analysis; MIBI-TOF, multiplexed ion beam imaging by time-of-flight; MPH, median pulse height; SOM, self-organizing map; TB, tuberculosis; TME, tumor microenvironment; TNBC, triple negative breast cancer.

CODEX relies on DNA barcoding of antibodies instead of heavy metals and more traditional fluorescent microscopy techniques (90, 91). DNA-barcoded antibodies are iteratively hybridized to fluorescently-tagged nucleotides in order to circumvent the issues of spectral overlap. After imaging, the fluorophores are then stripped (90, 91). This multiplexing method allows for the detection of over 50 DNA-conjugated antibodies in a single tissue section (92). Similar to other spatial proteomic technologies, CODEX analysis methods are still in active development, and the Nolan lab at Stanford has pioneered many of the tools currently available for processing CODEX data (https://github.com/nolanlab/CODEX) (8, 93). While both the mass-based approaches and CODEX have unique analysis pipelines, the broad steps necessary for interpretation of data are similar across both approaches (Figure 5).

### Preprocessing of Spatial Proteomics Data

MIBI-TOF preprocessing consists of two steps: image compensation and image normalization. After applying a Gaussian blur, MIBI-TOF images (often called fields of view) can be processed to remove the most common sources of background noise: gold ions from the conductive slide, organic hydrocarbons (noodles), isotopic impurities, and elemental contamination. Images are then normalized to account for the loss of mass spectrometry sensitivity that occurs during data acquisition on the mass spectrometer. Normalization and image compensation can be done using toffy, which integrates the Rosetta algorithm for image compensation and assesses detector sensitivity for image normalization using median pulse height.

CODEX data are preprocessed using the CODEX Toolkit Uploader (https://github.com/nolanlab/CODEX) (8). In short, this software concatenates and drift-compensates images, removes out-of-focus light using Microvolution (https://www.microvolution.com/), subtracts background, and creates hyperstacks of all fluorescence channels and imaging cycles (8). Background subtraction is performed using blank images without fluorescent oligonucleotides.

### Cell Segmentation

In multiplexed imaging data, each measurement represents a pixel rather than a single cell. Therefore, to obtain single-cell data similar to flow or mass cytometry, pixels must be aggregated and segmented into cells by identifying the border of each cell. Accurate cell segmentation is particularly important for all downstream analysis steps. The Angelo lab has developed Mesmer for MIBI-TOF, a deep learning algorithm trained on TissueNet with increased accuracy over previous analysis methods (94). Inputs to Mesmer are a membrane/cytoplasmic marker and a nuclear marker, which should be expressed across all cell types (95). Alternatively, multiple nuclear or membrane markers can be used to accommodate a wider variety of cell types. After segmentation, cells should be normalized for their respective areas since the fraction of the cell being represented can vary depending on how the tissue was cut as well as the cell type. The CODEX toolkit segmenter (https://github.com/nolanlab/CODEX) is used for CODEX data and requires a nuclear marker with the option of including a membrane marker. Spatial fluorescence compensation can also be performed with this tool.

### Clustering

After cell segmentation, MIBI-TOF data can be analyzed on either a pixel or cell level. While the cell level provides results similar to those from cytometric analysis of dissociated cells, and therefore can be used to phenotype cells, pixel-level data can capture information outside of cells and help better define cells that are close in space. Pixie (https://github.com/angelolab/ark-analysis) is a package available for both pixel- and cell-level iterative clustering based on the FlowSOM algorithm (96). For both types of clustering, Pixie provides cluster consistency scores to assess the quality of the clustering over many iterations. Clusters can be manually adjusted using Mantis Viewer (https://github.com/CANDELbio/mantis-viewer), which allows investigators to visualize the overlay of cell segmentation with cluster assignments and protein expression. After clustering, cells can be functionally characterized based on their protein expression profiles or quantified by their count, frequency, or density. The appropriate method of cell quantification is based on the characteristics of the tissue and the biological question.

CODEX data have mostly been analyzed via traditional cell clustering. Some common clustering methods for CODEX data include X-shift clustering with either angular or Euclidean distance, *k*-means clustering, and the Leiden community detection algorithm (93). The former methods are available in the package VorteX (https://github.com/nolanlab/vortex).

### Local Cell–Cell Interaction Analysis

Pairwise enrichment analysis is useful for creating a roadmap of cell–cell interactions. This method evaluates the spatial distance between two cell types by determining if the cells are found together more frequently than what would be expected by random chance (96). To increase the utility and specificity of this method, it is possible to make a context-dependent null distribution based on prior knowledge about the tissue architecture. For example, lymph nodes are known to have B cell follicles and T cell zones that are enriched for B and T cells, respectively. Additionally, instead of testing for enrichment relative to other cells, cells can be tested for enrichment relative to tissue features such as blood vessels, glands, etc.

Another method of evaluating local cell–cell interactions involves the generation of a SpatialScore, which has been pioneered on CODEX data (97). This method evaluates the distances between three cell types (C1, C2, and C3) by calculating a distance ratio of the minimal distances between C1–C2 (right distance) versus C1–C3 (left distance). C1 being significantly closer to either C2 or C3 defines the interactions between the cell types, and biological information can be used to determine how the results fit into a disease or functional context.

### Neighborhood-Based Analysis

While pairwise enrichment analysis is useful for looking at local cell–cell interactions, characterization of tissue microenvironments is a broader method for investigating the overall tissue architecture. This can be accomplished by either KNN analysis or spatial latent Dirichlet analysis (LDA). KNN is based on *k*-means clustering with the user inputs of the number of clusters and neighborhood size. As in clustering, microenvironments can be further characterized by counts or frequency depending on the biological question. There are a variety of KNN methods (https://github.com/nolanlab/NeighborhoodCoordination; https://github.com/angelolab/ark-analysis) available to evaluate neighborhoods in spatial proteomic data (98). Spatial LDA (https://github.com/angelolab/ark-analysis) has the same goal as KNN and requires the same user inputs. However, due to algorithmic differences, spatial LDA results in smoother changes between microenvironments and characterizes boundaries more effectively at the expense of computational power (96).

### Spatial Trajectory Inference

Trajectory inference methods for dissociated single-cell proteomics data can be applied to spatial proteomics datasets (99), but other methods incorporate spatial coordinates to perform spatial trajectory inference. SPATA2 (100) integrates calculated pseudotime or a user-defined trajectory with spatial coordinates to investigate gene or protein expression patterns along a spatial trajectory. stLearn (101) creates a pseudo-space-time distance metric for clusters of cells based on the spatial distance between clusters and pseudotime difference between cells within the clusters. The MST algorithm is used on the matrix of pseudo-space-time distance between clusters to create a rooted, directed tree connecting the clusters. Both of these methods were developed for use on spatial transcriptomics data but should be applicable to other spatial omics datasets as well, including spatial proteomics datasets.

## CONCLUSIONS

Analysis methods for mass cytometry and spatial proteomics data have greatly advanced in recent years, allowing larger, more complex datasets to be analyzed. While preprocessing of mass cytometry has become fairly standardized, downstream analyses and visualizations are highly dependent on the specific dataset and the biological questions under investigation. The breadth of biological questions researchers can answer with these datasets has expanded with advances in specific methods for DA and protein expression analyses, as well as trajectory inference. Beyond analyzing single cells in suspension, spatial proteomics has progressed in the last few years with the advent of CODEX, MIBI-TOF, IMC, and other related approaches. These technological developments have allowed for exciting new investigations into how cell–cell interactions and cell neighborhoods can influence tissue architecture and disease states. However, new spatial technologies present a challenge and opportunity for the development of new computational methods to meet the specific needs of spatial proteomic datasets. Tools have been developed for cell segmentation, pixel-level clustering, examining cell–cell interactions, and characterizing cell neighborhoods. The availability and usability of these tools are making spatial proteomic analysis more accessible and commonplace. We anticipate that, in the future, the continued expansion of well-documented and benchmarked analysis methods for both single-cell and spatial proteomics will further advance the discovery of novel biological insights that result from the use of these technologies.

## Figures and Tables

**Table 1:** Overview of batch correction methods in mass cytometry data

Method	Reference sample?	Clustering prior to normalization?	Approach	Normalization across multiple proteomics datasets?	Use case
CytofBatchAdjust	Yes	No	Scaling by factors (i.e., mean, median, percentile, or quantile)	No	Dorsal root ganglia from C57/BL6 mice (103)
CytofRUV	Yes	Yes	FlowSOM clustering followed by the RUV-III method (104) using pseudo-replicates	No	Chronic lymphocytic leukemia (105)
CytoNorm	Yes	Yes	FlowSOM clustering followed by quantile normalization using spline functions	No	NK cells from COVID-19 patients (106)
iMUBAC	No	Harmony prior to clustering	Unsupervised clustering followed by training of batch-specific cell type classifiers through machine learning	No	PBMCs from patients with OTULIN deficiency and healthy controls (107)
cyCombine	No	Marker standardization prior to clustering	Clustering using a SOM (108) followed by ComBat	Yes	Leukocytes in people with low antispike antibody levels after BNT162b2 vaccination (109)

Abbreviations: iMUBAC, integration of multibatch cytometry datasets; NK, natural killer; PBMC, peripheral blood mononuclear cell; RUV, remove unwanted variation; SOM, self-organizing map.

**Table 2:** Overview of trajectory inference algorithms

	Algorithm	Linear only?	User input(s)	Use case(s)
**Graph**	Diffusion map	No	Kernel width	B cells from PBMCs during immunotherapy for lung adenocarcinoma (human) (116)
	PAGA	No	Kernel for graph weight and partition (cell grouping)	CD4^+^ T cells from PBMCs (human) (56)
	SPADE	No	Number of clusters, outlier density cutoff, and target downsampling density	Neutrophil subsets during wound healing (117) and macrophage polarization during skeletal muscle regeneration (118) (both from mouse)
**Pseudotime**	CytoTree	No	Root cell(s) and (optionally) leaf cells (terminal cells for branches)	CD4^+^ T cells from splenocytes (human) (119)
	DPT	No	Kernel width (for diffusion map) and root cell	CD4^+^ T cells from PBMCs (human) (56) and lung adenocarcinoma tumor cells (human) (120)
	Monocle2	No	Root cell	β cells inT1D (human)^a^ (99)
	SCORPIUS	Yes	Number of clusters for *k*-means clustering^b^	Activation of naïve CD8^+^ T cells^c^ (121) and tetramer+ CD8^+^ T cells in chronic HBV (both from human) (122)
	Slingshot	No	Partition (cell grouping), root cluster, dim. red. (recommended), and (optionally) terminal clusters	CD4^+^ T cells from bone marrow (human) (123)
	Wanderlust	Yes	Root cell	Tetramer + CD8^+^ T cells during SARS-CoV-2 vaccination (mouse) (124) and stimulated CD8^+^ T cells in CVID (human) (125)
	Wishbone	No	Root cell	NK cell maturation in AML (human) (126)

Abbreviations: AML, acute myeloid leukemia; CVID, common variable immunodeficiency; dim. red., dimensionality reduction; PBMC, peripheral blood mononuclear cells; SARS-CoV-2, severe acute respiratory syndrome coronavirus 2; T1D, type 1 diabetes.

a Data generated with imaging mass cytometry and SCORPIUS was also used for trajectory inference.

b Default is *k* = 4.

c Also used Slingshot.

## BEST PRACTICES FOR EXPERIMENTAL DESIGN

Before starting an experiment, it can be helpful to follow best practices for experimental design in order to avoid technical artifacts and batch effects from influencing downstream analyses.

### Plate Design

Adding a reference sample across multiple batches can help to identify and remove shifts in signal due to technical variability. The reference sample should resemble the samples of interest as closely as possible, and all cell populations of interest should be represented. Furthermore, when comparing different groups or conditions to each other (e.g., healthy versus disease, treatment versus no treatment), the samples from each group should be distributed evenly across batches (and barcode plates) to ensure any batch variability has the same impact on all groups. Finally, for longitudinal studies whose primary goal is to investigate changes across time within each individual, all samples from the same participant should be placed on the same plate/batch to minimize the influence of batch effects on within-patient comparisons.

### Panel Design

Proper panel design can minimize spillover between different heavy metal isotope or fluorophore signals. Compensation can be used if signals from different channels overlap. This topic has been reviewed more thoroughly by Rybakowska et al. (18). Additionally, ensuring panel consistency across an experiment by making enough reagent aliquots for all batches and running all batches on consecutive days can minimize batch effects and downstream analysis issues.

## AUTOMATING THE PREPROCESSING STEPS AND ANALYSIS WORKFLOWS

A comprehensive overview of bioinformatic tools for preprocessing and analysis steps of single-cell proteomics data has been provided by Rybakowska et al. (18). However, many tools are implemented through different software or platforms that can complicate the analysis and harm reproducibility. Some packages, such as FlowClean (110), flowStats (https://github.com/RGLab/flowStats), and CATALYST (111, 112) (https://github.com/HelenaLC/CATALYST), can automate preprocessing steps. Additionally, cytoBank (14, 15), cytofkit (113), and Cyto (114) facilitate semiautomatic workflows for downstream analysis without the need for advanced computational skills. Another comprehensive workflow developed by Olsen et al. (115) is available at http://cytof.biosurf.org. For an R-based pipeline, we recommend cytofWorkflow (113), which utilizes CATALYST and other R/Bioconductor packages to streamline mass cytometry data analysis.

## FUTURE ISSUES

1. Further development and use of algorithms that resolve the use of median protein expression for differential protein expression analyses would improve reliability.
2. Dimensionality reduction methods that utilize machine learning approaches should be developed and utilized more broadly.
3. Trajectory inference tools are not as well benchmarked on mass and flow cytometry datasets as single-cell RNA sequencing tools.
4. While computational methods for spatial proteomics data have improved greatly, continued advancements are necessary for issues such as cell calling at adjacent cell membranes.
5. The rapid expansion of spatial proteomic technologies has led to a concurrent expansion in computational tools that are neither standardized nor always compatible across technologies. Robust pipelines that can be easily adapted to various spatial proteomic technologies would greatly increase utility of these computational tools.
